# Impact of Drp1 Loss on Organelle Interaction, Metabolism, and Inflammation in Mouse Liver

**DOI:** 10.3390/cells14100679

**Published:** 2025-05-08

**Authors:** Lixiang Wang, Seiji Nomura, Nao Hasuzawa, Sadaki Yokota, Ayako Nagayama, Kenji Ashida, Junjiro Rikitake, Yoshinori Moriyama, Masatoshi Nomura, Ken Yamamoto

**Affiliations:** 1 Department of Medical Biochemistry, Kurume University School of Medicine, Kurume 830-0011, Japan; nomura_seiji@kurume-u.ac.jp (S.N.); yamamoto_ken@med.kurume-u.ac.jp (K.Y.); 2 Division of Endocrinology and Metabolism, Department of Internal Medicine, Kurume University School of Medicine, Kurume 830-0011, Japan; hasuzawa@med.kurume-u.ac.jp (N.H.); nagayama_ayako@kurume-u.ac.jp (A.N.); ashida@med.kurume-u.ac.jp (K.A.); rikitake_junjirou@med.kurume-u.ac.jp (J.R.); moriyama_yoshinori@med.kurume-u.ac.jp (Y.M.); 3 Division of Functional Morphology, Faculty of Pharmaceutical Sciences, Nagasaki International University, Sasebo 859-3243, Japan; syokota123@gmail.com

**Keywords:** dynamin-related protein 1, lipid metabolism, organelle interaction, liver inflammation

## Abstract

Dynamin-related protein 1 (Drp1) is a crucial player in mitochondrial fission and liver function. The interactions between mitochondria, endoplasmic reticulum (ER), and lipid droplets (LDs) are fundamental for lipid metabolism. This study utilized liver-specific Drp1 knockout (*Drp1*LiKO) mice to investigate the effects of Drp1 deficiency on organelle interactions, metabolism, and inflammation. Our analysis revealed disrupted interactions between mitochondria and LDs, as well as altered interactions among ER, mitochondria, and LDs in *Drp1*LiKO mice. Through mass spectrometry and microarray analysis, we identified changes in lipid profiles and perturbed expression of lipid metabolism genes in the livers of *Drp1*LiKO mice. Further in vitro experiments using primary hepatocytes from *Drp1*LiKO mice confirmed disturbances in lipid metabolism and increased inflammation. These findings highlight the critical involvement of Drp1 in regulating organelle interactions for efficient lipid metabolism and overall liver health. Targeting Drp1-mediated organelle interactions may offer potential for developing therapies for liver diseases associated with disrupted lipid metabolism.

## 1. Introduction

Membrane contact sites are specialized regions where the membranes of two organelles are closely juxtaposed, typically within 10 to 80 nanometers, without fusion [1]. These sites are stabilized by tethering proteins that bridge the gap between organelles, forming a dynamic “social network” within the cell that coordinates and facilitates metabolic and other cellular functions [2]. Recent advances in these contact sites research have revolutionized our understanding of cell biology [3]. Rather than focusing solely on the individual functions of organelles, such as mitochondria (Mt) in β-oxidation or the endoplasmic reticulum (ER) in lipid synthesis and lipid droplet (LD) formation, there is growing attention on the cooperative interactions and communication between organelles. This emerging perspective highlights the critical role of the inter-organelle “social network” in maintaining cellular homeostasis, supporting development, and mediating responses to disease [4,5].

Among the diverse types of contact sites, interactions between mitochondria and other organelles, particularly the ER and LDs, have attracted significant interest as key regulators of cellular lipid metabolism [6,7]. The Mt–ER interface, mediated by mitochondria-associated ER membranes (MAMs), serves as a critical platform for various cellular processes, including autophagy, lipid transfer, apoptosis, and inflammation [8,9,10]. Similarly, Mt–LD contact sites are essential for fine-tuning lipid storage and utilization. These dynamic interfaces integrate energy production and lipid metabolism [11,12]. At these contact sites, fatty acids are directly transferred from LDs to mitochondria for β-oxidation, generating energy. Additionally, mitochondria contribute to lipid droplet biogenesis by supplying lipid precursors during energy-rich conditions, promoting triacylglycerol (TAG) storage. By balancing lipid storage in LDs and oxidation in mitochondria, these contact sites buffer cellular energy levels and minimize lipotoxicity by preventing lipid accumulation in non-LD compartments. Dysfunctions in these contact sites have been linked to metabolic diseases and inflammatory processes, underscoring their importance in maintaining cellular health [10,13,14,15]. Furthermore, recent studies have identified three-way Mt–ER–LD contact sites, shedding light on their roles in maintaining lipid storage, homeostasis, and metabolic balance [16]. Freyre et al. showed that Mitoguardin 2 links mitochondria, the ER, and LDs and promotes LD expansion, suggesting a complex tri-organelle interaction to facilitate fatty acid metabolic channeling [17]. Guyard et al. uncovered a novel function of MAM in promoting LD biogenesis and growth at sites where the ER interacts with both the mitochondria and the LDs, which they named MAM-LD contact sites [18]. Najt et al. also revealed that mitochondria, the ER, and LD interactions compartmentalize hepatic fatty acid trafficking and metabolism [19].

Mitochondrial dynamics, regulated by a balance between fusion and fission, are fundamental to both mitochondrial and cellular functions [20,21]. Notably, Mt–ER contact sites play a pivotal role in regulating mitochondrial fission. These fission events are spatially coordinated at Mt–ER contact sites, where ER tubules wrap around mitochondria at fission points. This is followed by the recruitment of Dynamin-related protein 1 (Drp1), a key regulator of mitochondrial fission, which forms ring-like structures around the mitochondria. The GTPase activity of Drp1 induces conformational changes that culminate in mitochondrial membrane division [22,23]. Interestingly, our previous study demonstrated that Drp1 defects influence Mt–ER interactions by decreasing the number of these contact sites [24]. Furthermore, recent studies have shown that inhibiting the Drp1–filamin complex reduces hepatic lipid accumulation by enhancing Mt–LD interactions [25].

Despite these advances, the precise mechanisms by which Drp1 regulates the structural and functional dynamics of organelle interactions remain poorly understood. This study aims to investigate how Drp1-deficiency-induced changes in mitochondrial morphology affect interactions between mitochondria, the ER, and LDs. Specifically, we focus on elucidating the role of Drp1 in lipid metabolism and its contribution to liver inflammation. Utilizing *Alb-Cre^Tg^*^/+^ *Drp1^flox^*^/*flox*^ (*Drp1*LiKO) mice and matched controls (Conts) fed either a normal chow diet (NCD) or a high-fat diet (HFD), we employed electron microscopy, mass spectrometry, microarray analysis, as well as assessments of lipid metabolism and inflammatory responses in primary hepatocytes derived from *Drp1*LiKO mice. This study highlights how Drp1 deficiency disturbs organelle interactions and lipid metabolism, thereby contributing to liver inflammation.

## 2. Materials and Methods

### 2.1. Animals

*Drp1*LiKO and control (*Drp1^flox/flox^*, sibling littermates) mice were generated as described earlier [26]. These mice were housed in a standard specific-pathogen-free facility at a temperature range of 22–24 °C and a relative humidity of 50–60%, with a 12 h light/12 h dark cycle (lights off at 8:00 p.m.). They were provided ad libitum access to a normal chow diet containing 5.4% fat (CRF-1; Orient Yeast, Tokyo, Japan). For the high-fat diet study, 4-week-old mice were fed a diet comprising 24% fat (consisting of lard fat, providing 45% of total calories from fat, D12451; Research Diets, New Brunswick, NJ, USA) for a duration of 16–24 weeks. Mice were sacrificed using a humane method to minimize suffering. Specifically, euthanasia was performed by carbon dioxide (CO_2_) inhalation, followed by cervical dislocation to ensure death. Experiments were conducted between 10:00 and 11:00 a.m. following an overnight fasting period. Refeeding samples were collected between 14:00 and 15:00 after a 4 h refeeding period after a 16 h fast. Prior to any surgical procedure, mice were anesthetized using isoflurane inhalation anesthesia. All experiments were performed using male mice, in compliance with approved institutional guidelines and ethical approval obtained from the Ethics Committees of Kurume University School of Medicine (renewed annually with the latest approval date of 1 April 2024, and approval code: 2024-134).

### 2.2. Total RNA Isolation, Microarray Procedures, and Real-Time-PCR

Total RNA was isolated from mouse liver and primary mouse hepatocytes using the RNeasy Plus Mini Kit (QIAGEN, Hilden, Germany) following the manufacturer’s instructions. For microarray analysis, complementary RNA (cRNA) was amplified and labeled using the Low Input Quick Amp Labeling Kit (Agilent Technologies, Santa Clara, CA, USA). The cRNA was then hybridized to a 60K, 60-mer oligomicroarray (SurePrint G3 Mouse Gene Expression Microarray 8x60K v2; Agilent Technologies) as per the manufacturer’s guidelines. For real-time PCR assays, 1000 ng of total RNA was transcribed into first-strand complementary DNA (cDNA) utilizing the QuantiTect Reverse Transcription Kit (QIAGEN, Hilden, Germany) following the manufacturer’s protocol. The cDNA was subsequently employed for quantitative real-time PCR using the TB Green Premix Ex Taq II Master Mix (Takara, Tokyo, Japan), with the process monitored by the StepOne Plus Real-Time PCR system (Thermo Fisher Scientific, Waltham, MA, USA). The primer sequences for the chosen genes are listed in Supplementary Table S1, and the relative gene expression was normalized to *Gapdh* expression.

### 2.3. Western Blot Analysis

Fresh mouse liver tissue and primary mouse hepatocytes were homogenized in a Western lysis buffer composed of 20 mM Tris-HCl (pH 7.6), 150 mM NaCl, 2 mM ethylenediaminetetraacetic acid (EDTA), and 0.5% NP40, supplemented with protease and phosphatase inhibitors (Roche Applied Science, Mannheim, Germany). Subsequently, the samples were mixed with Laemmli sample buffer (Bio-Rad, Hercules, CA, USA) and subjected to heat treatment at 95 °C for 5 min. Sodium dodecyl sulfate–polyacrylamide gel electrophoresis was conducted with an equal protein load from each sample. The specific primary and secondary antibodies used are detailed in Supplementary Table S2. Visualization of protein bands was achieved using Ez WestLumi plus (ATTO, Tokyo, Japan) and the ImageQuant LAS 4000 Western Blotting Detection System (Fujifilm, Tokyo, Japan).

### 2.4. Histology, Immunohistochemistry, and Electron Microscopy

The liver tissue was fixed by immersion in a 4% (*w*/*v*) paraformaldehyde (PFA) solution overnight at 4 °C. Subsequently, the fixed tissue was embedded in paraffin following standard protocols. Immunostaining was carried out by diluting primary and secondary antibodies in Can Get Signal™ Immunoreaction Enhancer Solutions A and B, respectively (Toyobo, Osaka, Japan). The primary and secondary antibodies used are listed in Supplementary Table S2. Hepatocyte apoptosis was assessed using the TUNEL assay kit (Burlington, Boston, MA, USA) according to established procedures. Tissue sections were observed using a BZ-8000 microscope (Keyence, Osaka, Japan). To minimize variability, quantitative analysis of F4/80-positive cells, TUNEL-positive cells, and necrotic cells was conducted by counting cells in a composite image generated using Keyence BZ-X Analyzer software. The composite image combined 15 high-power fields (20× magnification) to form a new joint image. Three joint images were analyzed per mouse, with 3–4 mice per experimental group.

For electron microscopy analysis, liver samples were obtained from overnight fasted mice between 14:00 and 15:00. Cytochrome oxidase and Glucose-6-Phosphate cytochemistry assays were conducted using the alkaline 3,3′-diaminobenzidine (DAB) procedure as previously described [27,28,29]. Briefly, thin sections of the liver tissues were prepared for electron microscopy analysis. The sections were fixed, dehydrated, embedded in resin, and then cut into ultrathin sections using an ultramicrotome. The ultrathin sections were treated with a solution containing DAB and the specific substrate for each enzyme for Cytochrome oxidase and Glucose-6-Phosphate. Following incubation with the DAB substrate, the sections were then incubated in the presence of hydrogen peroxide. After the enzymatic reaction developed, the sections were post-fixed using osmium tetroxide to enhance contrast and preserve the structure of the samples. Finally, the sections were examined under an electron microscope (model H7650, Hitachi; Tokyo, Japan). Morphometric measurements of mitochondria and lipid droplet sizes were performed using the ImageJ software (version 3), based on a minimum of five electron micrograph sections per mouse.

### 2.5. Lipidomics

The livers were promptly excised and flash-frozen in liquid nitrogen prior to homogenization using a MultiBeads Shocker. Lipid extraction was then carried out using a mixture of methanol and chloroform, followed by vigorous vortexing for 10 min and subsequent centrifugation at 1000× *g* for 15 min at room temperature. The lower organic phase was isolated and dried using a TurboVap LV system (Biotage, Tokyo, Japan). The resulting dried lipid extract was reconstituted in acetonitrile along with an internal standard for liquid chromatography–mass spectrometry (LC-MS) analysis. For the analysis, an Agilent 1260 HPLC system was coupled with an Agilent 6520 quadrupole time-of-flight (Q-TOF) mass spectrometer. The identification and curation of annotated lipids were performed using the Agilent MassHunter Profinder software (MasterHands ver.2.9.0.9). The relative concentrations of the detected metabolites are listed in Supplemental Table S3.

### 2.6. Isolation of Primary Mouse Hepatocytes, Cell Culture, and Imaging

In this study, we utilized a rapid two-step method to isolate primary hepatocytes from mice [30]. Palmitic acid and oleate (Sigma, St Louis, MO, USA) were initially dissolved in alcohol to achieve a final concentration of 0.2 M. Subsequently, the solution was further diluted in DMEM medium supplemented with 10% fatty acid-free BSA (Sigma, St Louis, MO, USA) to generate a 4 mM stock solution. For live-cell imaging experiments, the isolated hepatocytes were plated on a type I-coated 35 mm glass-based dish (Iwaki, Tokyo, Japan) and treated with MitoTracker^®^ Red and HCS LipidTox Green Neutral Lipid Stain following standard protocols (Thermo Fisher Scientific, Waltham, MA, USA). Subsequently, the hepatocytes were visualized using a confocal microscope LSM700 (Zeiss, Oberkochen, Germany). Fluorescence intensity profiling analysis was conducted using the ZEN lite software (Zeiss, Oberkochen, Germany) in accordance with the manufacturer’s guidelines.

### 2.7. Measurement of Cytokine Levels in Cell Culture Supernatants

Cytokine levels in primary mouse hepatocyte culture supernatants were quantified using the BD Cytometric Bead Array (CBA) Cytokine Kit (Becton Dickinson, Franklin Lakes, NJ, USA), NovoCyte Flow Cytometer (ACEA Biosciences, San Diego, CA, USA), and NovoExpress ver. 1.2.5 software.

### 2.8. Statistics and Reproducibility

Data were presented as mean ± standard error of the mean (SEM). Multiple group comparisons were performed using two-way ANOVA with Tukey’s post hoc test or Sidak’s post hoc test. Statistical analyses were carried out using GraphPad Prism 9.0 software (GraphPad, San Diego, CA, USA). Statistical significance was defined as *p* < 0.05, *p* < 0.01, and *p* < 0.001.

### 2.9. Data Availability

The microarray data discussed in this study have been deposited in the Gene Expression Omnibus (GEO) database (http://www.ncbi.nlm.nih.gov/geo/, accessed on 1 May 2025) under accession numbers GSE296127 and GSE296128. The data supporting the findings of this research are available from the corresponding authors upon reasonable request.

## 3. Results

### 3.1. Drp1 Deficiency Increases Interactions Between Mitochondria and Lipid Droplets

**Figure 1:**
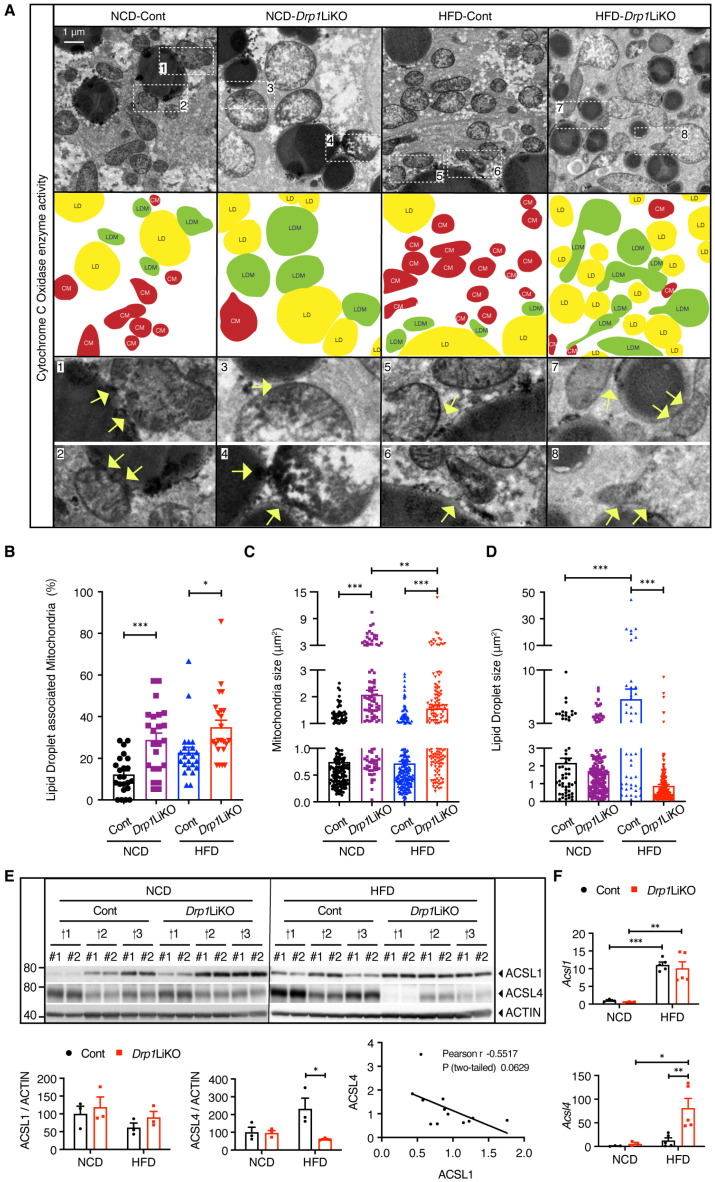
Increased mitochondria–lipid droplet interactions in *Drp1*LiKO mice. (**A**) Transmission electron microscopy images depicting cytochrome c oxidase enzyme activity assay in the livers of control and *Drp1*LiKO fasted mice. Regions denoted by white dashed squares are magnified in the lower panel. Lipid droplet-associated mitochondria (LDMs) are indicated in green, cytosolic mitochondria (CMs) in red, and lipid droplets (LDs) in yellow. Yellow arrows indicated mitochondria–lipid droplet interaction sites. Scale bar = 1 μm. (**B**–**D**) Quantitative analysis of the percentage of lipid droplet-associated mitochondria (*n* = 23), mitochondria size (*n* = 105–205), and lipid droplet size (*n* = 43–198) in control and *Drp1*LiKO fasted mice. Analysis was performed on a total of 23 images, with 3 mice per group. Values are expressed as mean ± SEM. * *p* < 0.05, ** *p* < 0.01, *** *p* < 0.001. Determined by two-way ANOVA with Tukey’s multiple comparisons test. (**E**) Western blot analysis, corresponding densitometric quantification, and correlation analysis of ACSL1 and ACSL4 expression levels. ACTIN served as the loading control. Technical duplicates labeled as #1 and #2; biological replicates labeled as †1, †2, and †3. Data presented as mean ± SEM (*n* = 3). * *p* < 0.05, based on two-way ANOVA with Tukey’s multiple comparisons test. (F) Real-time PCR evaluation of *Acsl1* and *Acsl4* mRNA expression levels. *Gapdh* employed as the internal control. Data displayed as mean ± SEM (*n* = 3–5). * *p* < 0.05, ** *p* < 0.01, *** *p* < 0.001. Based on two-way ANOVA with Tukey’s multiple comparisons test.

Mitochondria were classified based on their proximity to lipid droplets into LD-associated mitochondria (LDM, also known as peridroplet mitochondria) and cytosolic mitochondria (CM) [31,32]. Electron microscopy of cytochrome c oxidase enzyme activity assays allowed clear visualization of Mt–LD interactions (Figure 1A, indicated by yellow arrows). Quantitative analysis has shown a higher proportion of LDM in both NCD- and HFD-*Drp1*LiKO mice under fasting conditions, indicating increased Mt–LD interactions (Figure 1B). Additionally, an increase in mitochondrial size was observed in both NCD- and HFD-*Drp*1LiKO mice (Figure 1C). Notably, while lipid droplet size was larger in HFD-control mice, this enlargement was not present in HFD-*Drp1*LiKO mice (Figure 1D). The enzyme long-chain fatty acyl-CoA synthetase 1 (ACSL1) localizes to the outer mitochondrial membrane and interacts with lipid droplet-tethering proteins [33]. While ACSL4 is a marker of MAMs (Figure 1E), in *Drp1*LiKO mice, levels of ACSL1 protein remained unchanged; however, ACSL4 protein levels were significantly decreased in HFD-*Drp1*LiKO mice. A negative correlation was observed between ACSL1 and ACSL4 protein levels, with a Pearson correlation coefficient of −0.5517, indicating a potential interaction between these two proteins in cellular pathways (Figure 1E). Additionally, mRNA expression levels showed no change for *Acsl1* but significantly higher levels for *Acsl4* in HFD-*Drp1*LiKO mice compared to HFD-control mice, indicating that the decreased ACSL4 protein levels were not due to transcriptional regulation (Figure 1F).

### 3.2. Drp1 Deficiency Disrupts Interactions Among Mitochondria, the ER, and LDs

**Figure 2:**
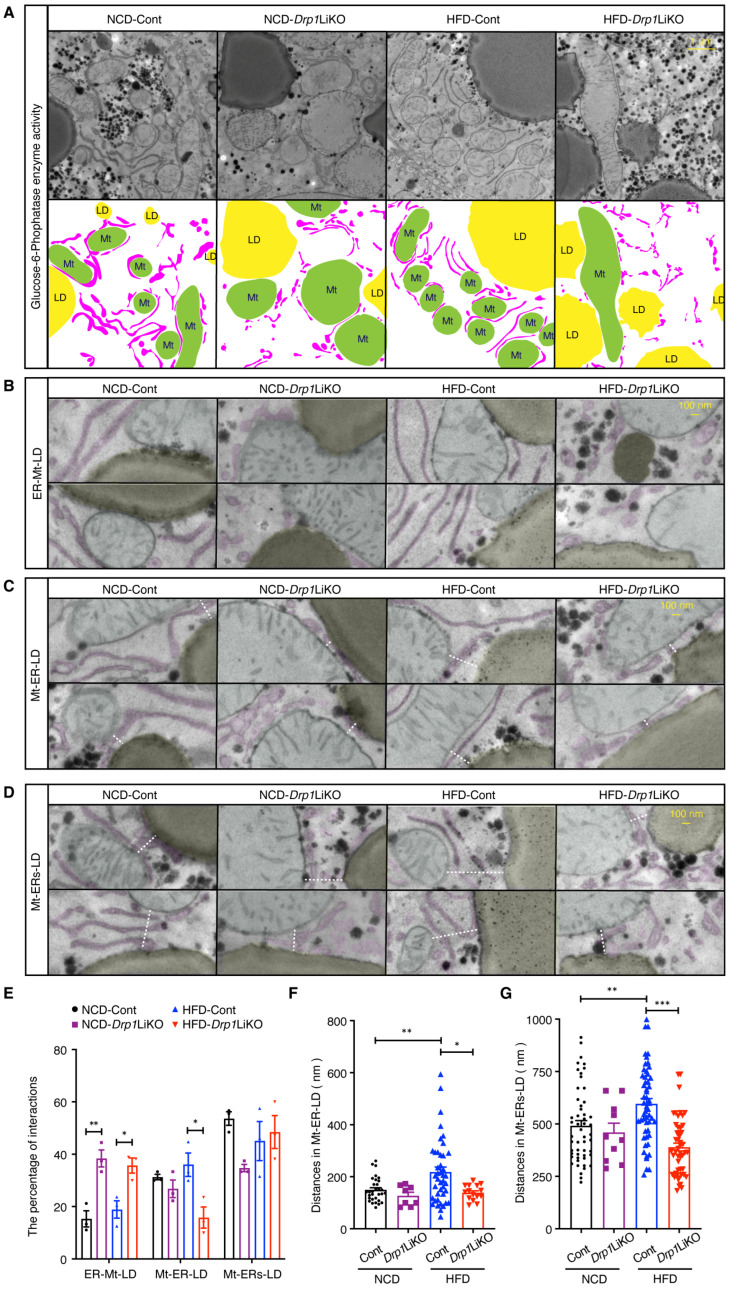
Disrupted interactions among mitochondria, the endoplasmic reticulum, and lipid droplets in *Drp1*LiKO mice. (**A**–**D**) Electron microscopy images showing glucose-6-phosphatase enzyme activity assay in the livers of control and *Drp1*LiKO mice. Mitochondria (Mt) are marked in green, lipid droplets (LDs) in yellow, and endoplasmic reticulum (ER) tubules in magenta. Dashed lines indicate the distances of the interactions among Mt, the ER, and LD. Scale bar = 1 μm in (**A**) and 100 nm in (**B**–**D**). (**E**–**G**) Quantitative analysis of the percentage (**E**) (*n* = 3) and distances (**F**–**G**) (*n* = 8–53) of interactions among Mt, the ER, and LD in control and *Drp1*LiKO fasted mice, with 3 mice per group. Values are expressed as mean ± SEM. * *p* < 0.05, ** *p* < 0.01, *** *p* < 0.001. Determined by two-way ANOVA with Tukey’s multiple comparisons test.

Najt et al. (2023) reported that interactions between mitochondria and the ER are crucial for regulating Mt–LD interactions [19]. In our Drp1-deficient models, a reduction in Mt–ER contacts [24] and an increase in Mt–LD interactions were observed. To investigate the relationships among mitochondria, lipid droplets (LDs), and the ER in *Drp1*LiKO mice, we performed a glucose-6-phosphatase enzyme activity assay, which stains the ER and allows clear visualization of organelle morphology using fasted mice. Electron microscopy revealed that, in control mice, the tubular ER frequently contacted and encircled mitochondria, likely limiting direct Mt–LD interactions. In contrast, *Drp1*LiKO mice exhibited a marked reduction in tubular ER, replaced by fragmented ER structures (Figure 2A). Najt et al. classified interactions among mitochondria, the ER, and LDs into two categories: ER–Mt–LD and Mt–ER–LD. In the ER–Mt–LD configuration (Figure 2B), mitochondria directly interact with the LD surface, with the ER wrapping around portions of the mitochondria. In the Mt–ER–LD configuration (Figure 2C), the ER is sandwiched between mitochondria and the LD, with no direct interaction between mitochondria and the LD. In our study, we observed cases where more than one ER tubule was positioned between mitochondria and the LD. Therefore, we further categorized Mt–ER–LD into two subtypes based on the number of ER tubules. Mt–ER–LD (Figure 2C): a single ER tubule is positioned between mitochondria and the LD. Mt–ERs–LD (Figure 2D): multiple ER tubules are positioned between mitochondria and the LD, with the distance between mitochondria and the LD being less than 1 μm. The percentage of ER–Mt–LD interactions increased in both NCD- and HFD-fed *Drp1*LiKO mice. In contrast, the percentage of Mt–ER–LD interactions decreased in HFD-*Drp1*LiKO mice (Figure 2E). The distances between mitochondria and LDs in Mt–ER–LD and Mt–ERs–LD (dashed white lines in Figure 2C,D) increased under a high-fat diet in control mice. However, this increase was absent in HFD-*Drp1*LiKO mice (Figure 2F,G).

### 3.3. Drp1 Deficiency Leads to an Altered Lipid Profile

**Figure 3:**
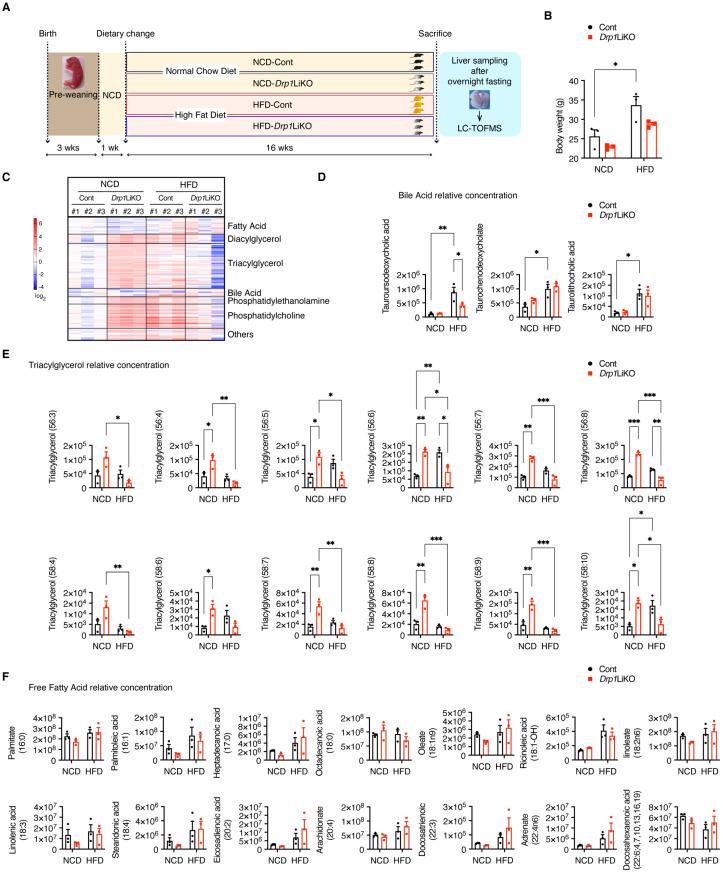
Aberrant lipid metabolite profiles in *Drp1*LiKO mice. (**A**) Schematic representation of the experimental procedures. (**B**) Body weights of the mice utilized in this study. Values expressed as mean ± SEM. *n* = 3. * *p* < 0.05. Determined by two-way ANOVA with Tukey’s multiple comparisons test. (**C**) Heatmap of lipid profiling. A heatmap was generated from the LC-MS analysis of liver lipid extracts. Biological replicate samples in each condition are numbered as #1, #2, and #3. (**D**–**F**) Relative concentrations of specific lipid species in the livers of control and *Drp1*LiKO mice. The quantities of triacylglycerol and free fatty acids are categorized by total carbon atoms and the number of double bonds. Values expressed as mean ± SEM. *n* = 3. * *p* < 0.05, ** *p* < 0.01, *** *p* < 0.001. Determined by two-way ANOVA with Tukey’s multiple comparisons test.

The interplay between mitochondria, ER, and LD plays a crucial role in maintaining cellular lipid balance between TAG and free fatty acids (FFA). In a previous study, we found that *Drp1*LiKO mice showed consistent levels of total TAG, total cholesterol, and total FFA in the liver when compared to control mice, under both fasting and refeeding conditions with a high-fat diet [24]. To gain further insight into the influence of Drp1-mediated organelle interactions on lipid metabolism, we conducted LC-MS analysis to examine the lipid profiles of both control and *Drp1*LiKO mice under fasting conditions, on NCD and HFD. We identified 15 lipid classes and 173 distinct lipid species and subsequently analyzed them using heatmaps and quantitative assessments. Figure 3A illustrates a flowchart of the experimental procedures, while the body weights of the mice involved are shown in Figure 3B. The heatmap data revealed distinctive lipid composition patterns in *Drp1*LiKO mice compared to the control group (Figure 3C). The relative concentrations of bile acids (Figure 3D and Supplementary Figure S1A), TAG (Figure 3E and Supplementary Figure S1B), and FFA (Figure 3F and Supplementary Figure S1C) were further examined. Notably, the levels of tauroursodeoxycholic acid, an ER stress inhibitor, were significantly higher in HFD-fed control mice compared to HFD-fed *Drp1*LiKO mice. Interestingly, while TAG levels increased in control mice under HFD conditions, the *Drp1*LiKO mice demonstrated significantly higher TAG levels under NCD and significantly lower levels under HFD. FFA levels, however, did not exhibit significant changes.

### 3.4. Drp1 Deficiency and Its Impact on Gene Expression in Lipid Metabolism

**Figure 4:**
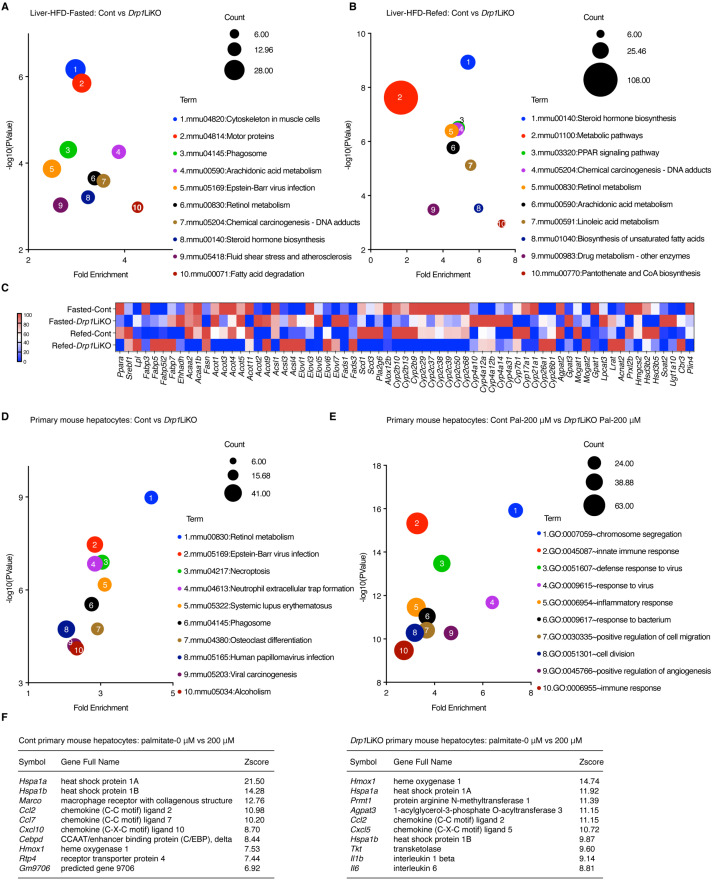
Aberrant gene expression profiles in HFD-*Drp1*LiKO mice and *Drp1*LiKO primary hepatocytes. (**A**,**B**) KEGG pathway enrichment analysis was conducted on differentially expressed genes in the livers of control and *Drp1*LiKO mice. The *X*-axis indicates the fold enrichment, while the *Y*-axis shows the negative logarithm (base 10) of the *p*-values. The size of the bubbles corresponds to the number of genes. (**C**) A heatmap illustrating differentially expressed genes associated with lipid metabolism that were identified through microarray analysis. (**D**) KEGG pathway enrichment analysis of differentially expressed genes in primary hepatocytes isolated from control and *Drp1*LiKO mice. (**E**) Gene ontology analysis of differentially expressed genes was performed on control and *Drp1*LiKO mouse primary hepatocytes treated with 200 μM palmitate. The *X*-axis represents fold enrichment, and the *Y*-axis denotes the negative logarithm (base 10) of *p*-values. The size of the bubbles corresponds to the gene count. (**F**) Top ten upregulated genes in control and *Drp1*LiKO hepatocytes following treatment with 200 μM palmitate, identified through microarray analysis.

To analyze the perturbations in lipid metabolism pathways in *Drp1*LiKO mice livers and primary hepatocytes, we performed Kyoto Encyclopedia of Genes and Genomes (KEGG) pathway enrichment analysis using the GSE296128 and GSE296127 dataset. In livers, under fasting and refed conditions, 32 and 35 pathways, respectively, exhibited significant enrichment (*p* < 0.05). Figure 4A,B illustrate the top 10 pathways, of which nine are directly associated with lipid metabolism. These pathways include arachidonic acid metabolism, retinol metabolism, steroid hormone biosynthesis, fatty acid degradation, metabolic pathways, peroxisome proliferator-activated receptor (PPAR) signaling, linoleic acid metabolism, biosynthesis of unsaturated fatty acids, and pantothenate and CoA biosynthesis. A heatmap of differentially expressed genes associated with lipid metabolism under fasting and refed states indicated pronounced changes in key signaling pathways (Figure 4C and Supplementary Figure S2). Subsequently, primary mouse hepatocytes from *Drp1*LiKO mice uncovered 57 KEGG pathways with significant enrichment, with the top 10 depicted in Figure 4D. Notably, retinol metabolism emerged as the most prominently enriched pathway. To further delineate the direct effects of these altered lipid metabolism pathways, we conducted gene ontology analysis on global gene expression profiles of primary hepatocytes treated with palmitate. The analysis revealed an upregulation of the innate immune response, inflammatory response, and immune response in Drp1-deficient cells (Figure 4E). Among the top ten upregulated genes following palmitate treatment, genes from the heat shock protein family, chemokine ligand family, and heme oxygenase were upregulated in both control and *Drp1*LiKO hepatocytes (Figure 4F). However, inflammatory cytokines interleukin-1β (*Il1b*) and interleukin-6 (*Il6*) were exclusively among the top ten genes upregulated in *Drp1*LiKO hepatocytes post-palmitate treatment.

### 3.5. Enhanced Inflammatory Response in Drp1LiKO Hepatocytes and Mice

**Figure 5:**
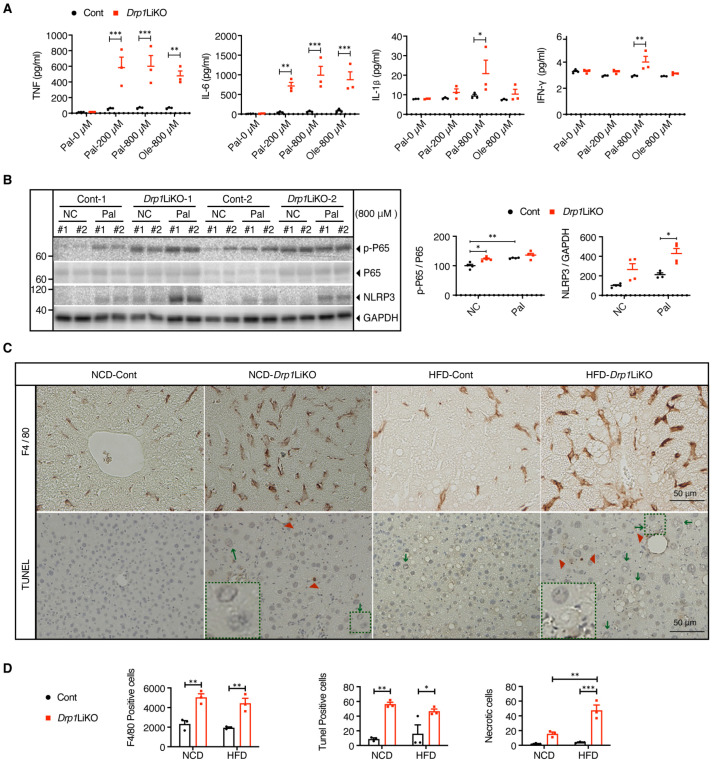
Inflammation and hepatocyte death in *Drp1*LiKO primary hepatocytes and mice. (**A**) Primary hepatocytes isolated from control and *Drp1*LiKO mice were treated with PBS (referred to as palmitate-0 μM), palmitate (200 or 800 μM), or oleate (800 μM) for 24 h. Cells and culture supernatants were collected. TNF, IL-6, IL-1β, and IFN-γ levels were determined using a BD cytometric bead array (*n* = 3). Values are expressed as mean ± SEM. * *p* < 0.05, ** *p* < 0.01, and *** *p* < 0.001 determined by two-way ANOVA with Sidak’s post hoc test. (**B**) Western blot analysis and densitometric quantification of p-P65 NF-κB, P65 NF-κB, and NLRP3 in PBS- or palmitate-treated cells. GAPDH served as the internal control. The technical duplicate samples in each condition were numbered as #1 and #2. Biological samples are numbered from Cont 1-2 and *Drp1*LiKO 1-2. Values are expressed as mean ± SEM (*n* = 4). * *p* < 0.05, ** *p* < 0.01 determined by two-way ANOVA with Tukey’s multiple comparisons test. (**C**,**D**) Control and *Drp1*LiKO mice were fed either an NCD or HFD for 16–24 weeks. Liver sections were subjected to F4/80 immunostaining and TUNEL assay to evaluate histological changes. (**C**) Representative images showing liver histology. TUNEL-positive cells indicated by red arrowheads, and necrotic cells indicated by green arrows. Areas indicated with green dashed squares are enlarged and displayed on the bottom left. Scale bar = 50 μm. (**D**) Quantitative analysis of F4/80-positive cells, TUNEL-positive cells, and necrotic cells performed by counting cells in composite images created by merging 15 high-power fields (20× magnification). Each mouse was analyzed using 3 joint images, with 3–4 mice per experimental group. Values expressed as mean ± SEM. *n* = 3–4. * *p* < 0.05, ** *p* < 0.01, *** *p* < 0.001. Determined by two-way ANOVA with Tukey’s post hoc test.

Since palmitate (16:0) and oleate (18:1) are the most abundant fatty acids in Western diets and rodent triglycerides, these fatty acids were used to further investigate the inflammatory response to FFAs in *Drp1*LiKO primary hepatocytes. We measured the levels of proinflammatory cytokines in the culture medium. Tumor necrosis factor (TNF), IL-6, IL-1β, and interferon-gamma (IFN-γ) were significantly elevated in *Drp1*LiKO hepatocytes compared to controls (Figure 5A). Consistently, FFA-treated *Drp1*LiKO hepatocytes showed increased mRNA expression of ER stress markers and proinflammatory cytokines (Supplementary Figure S3A). Western blot analysis confirmed activation of the P65 nuclear factor kappa B (NF-κB) and NLR family pyrin domain-containing 3 (NLRP3) inflammasome pathways, with stronger responses observed in *Drp1*LiKO hepatocytes (Figure 5B). Histological analysis of liver sections from NCD- and HFD-*Drp1*LiKO mice revealed clustered inflammatory cell infiltration, as shown in our previous study [24]. Immunostaining indicated that the predominant immune cell population was F4/80-positive macrophages (Figure 5C,D and Supplementary Figure S3B). Terminal deoxynucleotidyl transferase dUTP nick end labeling (TUNEL) assays demonstrated minimal apoptotic cells in control mice but a marked increase in *Drp1*LiKO mice. Additionally, necrotic cells, characterized by enlarged and vacuolated nuclei, were observed in NCD-*Drp1*LiKO mice and further increased in HFD-*Drp1*LiKO mice (Figure 5C,D). Collectively, these findings indicate that Drp1 deficiency exacerbates the inflammatory response, potentially contributing to hepatic dysfunction.

### 3.6. Mt–LD Interactions in Drp1LiKO Primary Hepatocytes

**Figure 6:**
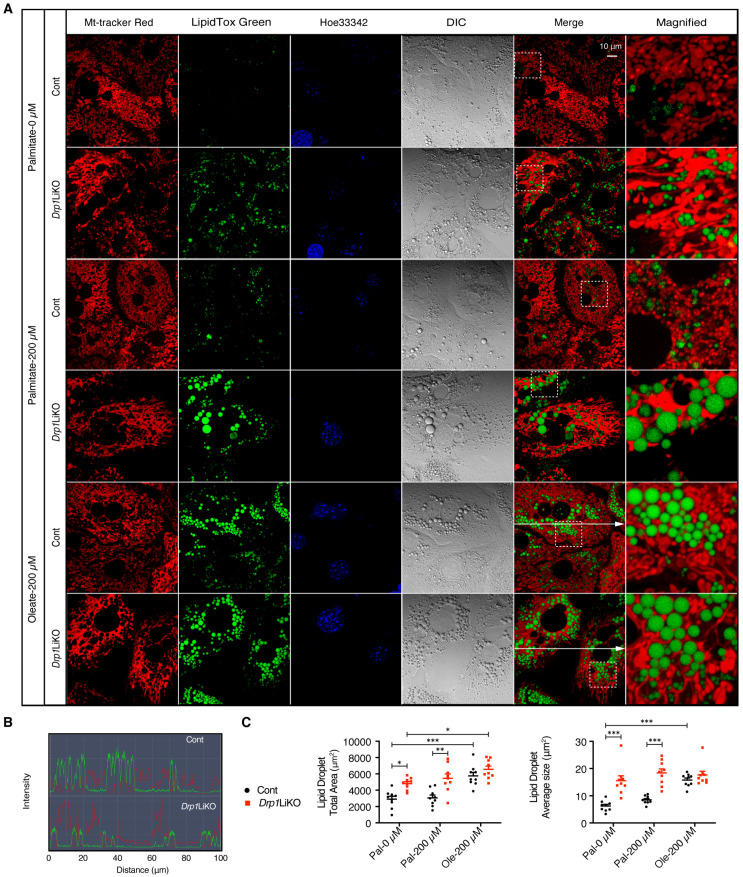
Increased lipid droplet formation in *Drp1*LiKO mouse primary hepatocytes. (**A**) Primary hepatocytes isolated from control and *Drp1*LiKO mice were treated with PBS (referred to as palmitate-0 μM), palmitate (200 μM), or oleate (200 μM) for 24 h. Representative images show MitoTracker Red staining for mitochondria and LipidTox Green for lipid droplets. Nuclei are stained with Hoechst 33342 (blue). Differential interference contrast (DIC) imaging enhances contrast in brightfield images. Areas indicated with white dashed squares are enlarged and displayed on the right. Scale bar = 10 μm. (**B**) Intensity profile analysis of pixels marked by the line shown in panel (**A**). (**C**) Quantitative analysis of the total area and average size of lipid droplets in the control and *Drp1*LiKO primary hepatocytes. Values are expressed as mean ± SEM. *n* = 9 (3 images from 3 individual experiments). * *p* < 0.05, ** *p* < 0.01, *** *p* < 0.001 determined by two-way ANOVA with Tukey’s post hoc test.

To investigate the spatial relationship between mitochondria and lipid droplets in vitro, live-cell imaging was conducted using MitoTracker Red to label mitochondria and LipidTOX Green to label lipid droplets. Remarkably, the study revealed that nearly all LDs were either in contact with short, fragmented mitochondria or were surrounded by long, tubular mitochondria, as illustrated in Figure 6A. The fluorescence intensity profile demonstrated that LDs were predominantly localized at the periphery of mitochondria in both control and *Drp1*LiKO hepatocytes (Figure 6B). Quantitative analysis indicated that the total LD area and the average size of LDs were significantly larger in *Drp1*LiKO hepatocytes compared to control cells, under both untreated and palmitate-treated conditions (Figure 6C).

## 4. Discussion

This study builds upon our previous investigations into the role of Drp1 in Mt–ER contact sites and liver function. In our initial study, we demonstrated that defects in Drp1 lead to a reduction in the number of Mt–ER contacts, which is associated with increased ER stress and activation of the unfolded protein response. This response in *Drp1*LiKO mice is characterized by elevated levels of phosphorylated eIF2α, resulting in translational inhibition [24]. We speculate that this translational inhibition might partly explain the observed reduction in ACSL4. Additionally, we showed that deletion of Drp1 does not significantly affect mitochondrial function, as evidenced by stable levels of oxygen consumption, ATP production, and mitochondrial respiratory complex protein levels. In our subsequent study, we found that liver-specific deletion of Drp1, as well as downregulation by siRNA in hepatocytes, induces liver inflammation. Furthermore, primary hepatocytes from *Drp1*LiKO mice exhibit a collapse of the mitochondrial membrane potential, which was found to be more prevalent [30].

**Figure 7:**
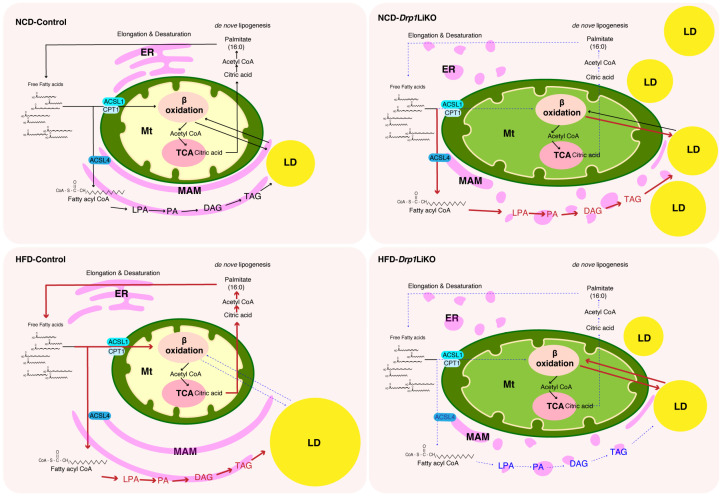
Disrupted lipid metabolism in the liver of *Drp1*LiKO mice and the potential impact of organelle interactions on lipid levels under varying dietary conditions. In control mice exposed to a high-fat diet (HFD), elevated levels of lysophosphatidic acid (LPA), phosphatidic acid (PA), diacylglycerol (DAG), and triacylglycerol (TAG) were observed. Conversely, NCD-*Drp1*LiKO mice exhibited significantly higher levels of LPA, PA, DAG, and TAG under normal chow diet (NCD) conditions but significantly lower levels under HFD conditions. The increased mitochondrial–lipid droplet (Mt-LD) interactions observed in NCD-*Drp1*LiKO mice may play a dual role as both a contributing factor and a consequence of the elevated TAG levels. Upon exposure to a high-fat diet, control mice showed an increase in lipid droplet size along with increased distances between mitochondria and lipid droplets in Mt-ER-LD and Mt-ERs-LD interactions. This spatial arrangement could potentially hinder lipid transfer between mitochondria and lipid droplets. In HFD-*Drp1*LiKO mice, the absence of increased distances between mitochondria and lipid droplets might be related to the observed reduction in TAG levels.

Our novel study reveals that Drp1 deficiency in the liver is associated with increased Mt–LD and ER–Mt–LD interactions, along with reduced Mt–ER–LD interactions. Previous studies accentuate the importance of Mt–LD interactions in fatty acid metabolism. For example, LDM in brown adipocytes with impaired fatty acid β-oxidation capacity can lead to lipid droplet expansion [31]. Conversely, in the liver, LDM might augment fatty acid oxidation while diminishing respiratory efficiency [32]. The increased Mt–LD interactions observed in NCD-*Drp1*LiKO mice might be both a cause and a consequence of elevated TAG levels (Figure 7). Mt–ER–LD contact sites are known to contain proteins facilitating lipid transfer between organelles [19]. Particularly noteworthy are the roles of proteins like ORP5 and ORP8, localized at Mt–ER–LD contact sites, in LD biogenesis and maintenance [18]. When exposed to a high-fat diet, control mice exhibited an increase in lipid droplet size and greater distances between mitochondria and lipid droplets in Mt–ER–LD and Mt–ERs–LD interactions. This spatial arrangement could prevent excessive triglyceride breakdown and limit the accumulation of deleterious fatty acids. In *Drp1*LiKO mice on a high-fat diet, the decreased Mt–ER–LD interactions may be associated with reduced TAG levels (Figure 7).

Mitochondrial morphology can modulate fatty acid utilization by affecting Cpt1 sensitivity, with elongated mitochondria enhancing sensitivity and fragmented mitochondria reducing it [34]. In *Drp1*LiKO mice, the absence of Drp1 altered mitochondrial morphology, which might impact fatty acid oxidation by modifying Cpt1 sensitivity to malonyl-CoA inhibition. In vitro studies confirmed these findings, as palmitate induced triglyceride accumulation within LDs and mitochondrial fragmentation in control hepatocytes, while oleate led to larger LD formation without inducing mitochondrial fragmentation, consistent with previous reports [35]. In contrast, *Drp1*LiKO hepatocytes exhibited larger and more numerous LDs even without fatty acid treatment, and post-treatment with palmitate or oleate further increased LD size and number, accompanied by tubular mitochondria. LDs play a vital role in managing excess free fatty acids, storing lipids, and alleviating ER stress by sequestering misfolded proteins, surplus lipids, and calcium [36,37,38]. Disruptions in LD formation, interactions between mitochondria and LDs, and mitochondrial fatty acid β-oxidation are pivotal in the development of metabolic dysfunction-related diseases, perpetuating lipid accumulation and metabolic dysfunction that exacerbate inflammation.

In this study, we highlight the significant role of intracellular organelle communication, termed the “social network”, in maintaining cellular function. Although we did not directly assess the role of these interactions in lipid metabolism and inflammation, our study highlights the intriguing topic of inter-organelle connectivity and its significance in maintaining cellular homeostasis. Further investigation is required to determine whether the heightened inflammation observed in *Drp1*LiKO mice results directly from disrupted organelle interactions or is a consequence of lipid accumulation. Additional experiments utilizing specific markers for Mt–LD contact sites are necessary. While proteins such as RAB8-PLIN5, MFN2-PLIN5, and ACSL1-SNAP23 have been identified as Mt–LD tether proteins, there is currently a lack of specific markers for Mt–LD contact sites. Future experiments involving the knockdown of these specific tether proteins in *Drp1*LiKO mice could shed light on whether reducing Mt–LD interactions enhances the outcomes associated with Drp1 deletion. Alternatively, restoring Mt–ER contacts through a linking molecule in *Drp1*LiKO mice might elucidate the effects on metabolic and inflammatory phenotypes, offering deeper insights into Drp1’s role in mitochondria, ER, and lipid droplet interactions and its impact on liver lipid metabolism and inflammation.

## 5. Conclusions

Metabolic dysfunction-associated steatohepatitis (MASH), previously termed nonalcoholic steatohepatitis (NASH), is a severe liver condition characterized by fibrosis, necroinflammation, and steatosis. As an advanced manifestation of metabolic dysfunction-associated steatotic liver disease (MASLD), MASH poses a significant global health challenge [39,40]. The prevalent lifestyle of excessive nutrient intake fosters chronic inflammation through metabolic byproduct accumulation, disrupting lipid metabolism and contributing to MASH development. In conclusion, our study, by employing a liver-specific Drp1 knockout mouse model, sheds light on MASH pathogenesis, elucidating how Drp1 deficiency exacerbates liver inflammation by disrupting organelle interactions and disturbing lipid metabolism. Targeting Drp1-related pathways presents novel therapeutic avenues for alleviating MASH. As research advances, restoring proper organelle communication and lipid balance may offer fresh strategies for managing metabolic-related disorders.

## Data Availability

The microarray data discussed in this study have been deposited in the Gene Expression Omnibus (GEO) database (http://www.ncbi.nlm.nih.gov/geo/, accessed on 1 May 2025) under accession numbers GSE296127 and GSE296128.

## Supplementary Materials

The following supporting information can be downloaded at: https://www.mdpi.com/article/10.3390/cells14100679/s1, Figure S1. Aberrant lipid metabolite profiles in *Drp1*LiKO mice. Figure S2. Signaling pathway of differentially expressed lipid metabolism-associated genes in *Drp1*LiKO mice. Figure S3. Inflammatory response in control and *Drp1*LiKO primary hepatocytes and mouse livers. Figure S4. Uncropped Western blot images for Figure 1E. Figure S5. Uncropped Western blot images for Figure 5B. Table S1. Real-time PCR primers used in this study. Table S2. Antibodies and regents used in this study. Table S3. Relative concentration of metabolites for lipidomics in control and *Drp1*LiKO mouse livers.

## Abbreviations

The following abbreviations are used in this manuscript:

ACSL	long-chain fatty acyl-CoA synthetases
CM	cytosolic mitochondria
DAG	diacylglycerol
Drp	dynamin-related protein 1
*Drp1*LiKO	liver-specific Drp1 knockout
ER	endoplasmic reticulum
FFA	free fatty acid
HFD	high-fat diet
LD	lipid droplet
LDM	LD-associated mitochondria
MAM	mitochondria-associated ER membrane
NCD	normal chow diet
NF-κB	nuclear factor kappa B
Nlrp3	NLR family pyrin domain-containing 3
TAG	triacylglycerol
TNF	tumor necrosis factor
TUNEL	terminal deoxynucleotidyl transferase dUTP nick end labeling
