# Supplementary material for: Impact of Drp1 Loss on Organelle Interaction, Metabolism, and Inflammation in Mouse Liver
Source: Cells. 2025 May 8;14(10):679. doi: 10.3390/cells14100679 (PMC12109831; doi:10.3390/cells14100679)
Supplement: Supplementary file 1 [file cells-14-00679-s001.zip › cells-3603396-supplementary.pdf]

Supplementary Material

Impact of Drp1 Loss on Organelle Interaction, Metabolism, and Inflammation in Mouse Liver

Lixiang Wang[1*], Seiji Nomura[1], Nao Hasuzawa[2], Sadaki Yokota[3], Ayako Nagayama[2], Kenji Ashida[2], Junjiro Rikitake[2], Yoshinori Moriyama[2], Masatoshi Nomura[2*] & Ken Yamamoto[1]

[1] Department of Medical Biochemistry, Kurume University School of Medicine, Kurume, 830-0011, Japan
[2] Division of Endocrinology and Metabolism, Department of Internal Medicine, Kurume University School of Medicine, Kurume, 830-0011, Japan
[3] Division of Functional Morphology, Faculty of Pharmaceutical Sciences, Nagasaki International University, Sasebo, Nagasaki, Japan

[*] **Corresponding authors:**
Masatoshi Nomura, MD PhD; Tel: +81-942-31-7563; Fax: +81-942-35-8943; E-mail: nomura@med.kurume-u.ac.jp
Lixiang Wang, MD PhD; Tel: +81-942-31-7544; Fax: +81-942-31-4377; E-mail: ourika0211@kurume-u.ac.jp

Contents

Supplementary Tables S1-S3

Supplementary Table S1. Real-time PCR primers used in this study.
Supplementary Table S2. Antibodies and regents used in this study.
Supplementary Table S3. Relative concentration of metabolites for lipidomics in control and *Drp1*LiKO mouse livers.

Supplementary Figures S1-S5

Supplementary Figure S1. Aberrant lipid metabolite profiles in *Drp1*LiKO mice.
Supplementary Figure S2. Signaling pathway of differentially expressed lipid metabolism-associated genes in *Drp1*LiKO mice.
Supplementary Figure S3. Inflammatory response in control and *Drp1*LiKO primary hepatocytes and mouse livers.
Supplementary Figure S4. Uncropped western blot images for Figure 1E.
Supplementary Figure S5. Uncropped western blot images for Figure 5B.

Supplementary Table S1. Real-time PCR primers used in this study.

ESM Table 1. Real-time PCR primers used in this study.

Species	Gene	Forward primer (5′ – 3′)	Reverse primer (5′ – 3′)	Product size (bp)
Murine	*Tnfa*	CCACCACGCTCTTCTGTCTA	AGGGTCTGGGCCATAGAACT	103
Murine	*Acsl1*	GTCCTGGGCACAGAAGAGAG	TGTTCCTGCACAGTTCTTCG	56
Murine	*Acsl4*	ACTGTCCCCTCAGACACACC	TGTTCCAGCACCACATGATT	94
Murine	*P8*	CAGACCACAGACACCACACC	TCTGCTTCTTGCTCCCATCT	222
Murine	*Chop*	AGCCTGGTATGAGGATCTGC	CTCCTGCTCCTTCTCCTTCA	319
Murine	*Il6*	CCGGAGAGGAGACTTCACAG	TTCTGCAAGTGCATCATCGT	166
Murine	*Gapdh*	CCATCACTGCCACCCAGAAG	GATGCAGGGATGATGTTC	91

Supplementary Table S2. Antibodies and regents used in this study.

ESM Table 2. Antibodies and reagents used in this study.

Antibodies and Reagents	Final Conc	Source	Material Number
Rat monoclonal anti F4/80	1:200	AbD serotec	MCA497
Rat monoclonal anti Ly6G	1:200	BD Biosciences	551459
Rat monoclonal anti-CD3	1:200	Biocompare	MCA500G
Rat monoclonal anti-CD45R/B220, clone RA3-6B2	1:200	BioLegend	103202
Rabbit polyclonal anti- pNFκB, p65 subunit (Ser536)	1:1000	Cell Signaling Technology	#3033
Mouse monoclonal anti-NFκB, p65 subunit, clone 12H11	1:1000	Merck	MAB3026
Mouse monoclonal anti-NLRP3/NALP3 (Cryo-2)	1:1000	Adipogen	AG-20B-0014
Rabbit polyclonal anti-Acsl1	1:1000	Cell Signaling Technology	#4047
Rabbit polyclonal anti-Acsl4	1:1000	Proteintech	22401-1-AP
Peroxidase-conjugated monoclonal anti-GAPDH	1:5000	Wako	015-25473
Peroxidase-conjugated monoclonal anti-β-ACTIN	1:5000	Wako	017-24573
Horse anti-mouse IgG, HRP-linked antibody	1:5000	Cell Signaling Technology	#7076
Goat anti-rabbit IgG, HRP-linked antibody	1:5000	Cell Signaling Technology	#7074
Goat anti-rat IgG, HRP-linked antibody	1:5000	Cell Signaling Technology	#7077
MitoTracker Red CMXRos	250 nM	Molecular Probes	M7512
HCS LipidTox Green Neutral Lipid Stain	1:1000	Thermo Fisher Scientific	H34475
Hoechst 33342	1 μg/ml	Dojindo	H342
ApopTag® Peroxidase In Situ Apoptosis Detection Kit		Milipore	S7100

Supplementary Table S3. Relative concentration of metabolites in control and *Drp1*LiKO mice livers.

Category	Name	NCD-W-1	NCD-W-2	NCD-W-3	NCD-K-1	NCD-K-2	NCD-K-3	HFD-W-1	HFD-W-2	HFD-W-3	HFD-K-1	HFD-K-2	HFD-K-3
Acyl carnitine	O-Stearoyl-L-carnitine(18:0)	165	134	108	195	138	244	182	196	290	290	184	292
Bile acid	Tauroursodeoxycholic acid	65498	95538	167414	123082	134344	111388	557746	1167092	900035	508028	363442	356148
Bile acid	Glycocholate	23396	51290	52117	7659	24636	25202	38102	63110	54224	38038	24768	28953
Bile acid	Taurocholate	11393652	14887410	20220979	10848046	12816914	17292202	12032648	16097109	10789376	14049436	11156300	10721758
Bile acid	α-Muricholic acid	297056	698726	750996	872120	108088	176267	205290	264938	154087	1111290	466706	633776
Bile acid	β-Muricholic acid	109493	246016	278623	315998	44398	69532	83230	107884	61847	396734	178684	222810
Bile acid	Cholic acid	65808	54691	690258	292272	93854	184183	70948	168094	36930	257036	28136	100709
Bile acid	Taurochenodeoxycholate	193362	510206	404468	676435	498442	638866	696664	1063302	1246088	1244522	1119693	911890
Bile acid	Glycodeoxycholate	0	0	24	0	14	0	16	13	12	18	0	12
Bile acid	Taurodeoxycholic acid	1095284	1684100	2711112	2005376	1832038	1258738	3972164	3989414	3174837	4431794	1156848	3305342
Bile acid	Ursodeoxycholic acid, Ursodiol	2846	3644	11512	6906	3155	986	16150	148334	69408	5358	6354	3940
Bile acid	Taurolithocholic acid	10810	17894	27564	32667	24974	13717	85236	149820	101044	142258	50644	108698
Bile acid	Chenodeoxycholate	8744	12536	9444	7977	2936	2534	13858	18288	11507	27648	5692	15341
Diacylglycerol	DG(32:0)	884	362	606	1969	4217	2974	1824	797	2195	1228	561	137
Diacylglycerol	DG(32:1)	3556	1562	1566	5512	14696	9147	10708	4633	17850	4107	2232	456
Diacylglycerol	DG(32:2)	2322	967	1556	3592	5562	4146	2780	1644	3643	1363	1130	302
Diacylglycerol	DG(34:1)	37575	12936	22801	68612	141284	93720	110239	51669	144942	59553	27716	4934
Diacylglycerol	DG(34:2)	100030	44340	70131	192766	331255	241640	237333	112382	278730	104136	57500	10046
Diacylglycerol	DG(34:3)	2143	1108	1107	3616	7909	5594	6329	2872	8656	2625	1475	251
Diacylglycerol	DG(36:2)	50594	10008	31593	80520	156034	92926	180384	90282	237258	95040	41214	7946
Diacylglycerol	DG(36:3)	126651	33686	94794	224528	369056	229200	310562	155734	331306	150679	75240	15104
Diacylglycerol	DG(36:4)	56652	17364	47406	93897	146902	84646	87684	49143	121335	42372	21080	4079
Diacylglycerol	DG(38:2)	2670	374	2554	5379	10730	4034	4288	2241	6994	2445	1076	175
Diacylglycerol	DG(38:4)	1916	404	1686	4958	9180	4175	6848	3911	10774	3804	1550	234
Diacylglycerol	DG(38:3)	214	760	254	1116	1722	1100	1650	834	0	843	1055	388
Diacylglycerol	DG(38:4)	5779	3150	6776	36691	46130	31468	57080	25798	86846	25467	9218	1138
Fatty Acid	Dodecanoic acid(12:0)	1031110	937600	783384	576272	780446	704198	742367	3881984	1670727	1946944	807211	1291053
Fatty Acid	5-Dodecenoate (12:1n7)	63564	74688	53172	71582	49642	59984	50102	46332	69941	65908	52244	36108
Fatty Acid	Myristic acid(14:0)	6691626	8742672	5173438	3228270	4780222	4631842	5330741	8031010	15611800	15955026	4459063	10961352

Fatty Acid	2-Hydroxytetradecanoic acid(14:0-OH)	71990	87926	77587	63285	76362	51953	25498	33425	91730	112132	32945	53923
Fatty Acid	Myristoleate (14:1n5)	731041	803367	438960	263243	543798	433028	264008	336021	915848	624660	298566	608148
Fatty Acid	Pentadecanoate (15:0)	216900	368367	197437	193204	208044	201830	150854	159261	347585	403592	147580	215480
Fatty Acid	Palmitate(16:0)	206782597	269896582	198773148	#########	#########	202858726	211360066	257027164	308433928	349242090	########	#########
Fatty Acid	Palmitoleic acid (16:1)	36998303	65886536	22814704	10961309	24255863	22412564	45441316	70685858	141667460	95202384	26989387	80335887
Fatty Acid	Heptadecanoic acid(17:0)	2354758	2100988	2329138	960621	881330	1780102	2368591	3463392	6428523	9231116	1579053	5502472
Fatty Acid	Octadecanoic acid(18:0)	82871678	95815874	90858236	#########	70718909	134434086	112748814	101248048	65878038	66832373	94351777	49342022
Fatty Acid	Oleate (18:1n9)	2439506	2716652	2145406	1349582	1726749	1668268	1554431	2404618	4154260	4690437	1431545	3453014
Fatty Acid	Ricinoleic acid(18:1-OH)	132646	149855	120720	166807	164067	177730	323990	342526	569454	439218	289238	295254
Fatty Acid	linoleate(18:2n6)	173108692	188273214	152917176	#########	#########	129167968	121576171	177171163	255168001	276653552	########	#########
Fatty Acid	Linolenic acid(18:3)	9773934	23728840	6779744	3107017	6664906	6100091	7787080	14139526	28586124	21829052	4205966	16493122
Fatty Acid	Stearidonic acid (18:4)	974334	2011874	571520	294140	606488	564758	1091613	1876939	5143264	4247278	884698	3551000
Fatty Acid	Eicosenoic acid (20:1)	3836650	1692429	3657352	1835512	2719421	2327142	2550610	2347924	1519874	2420569	1608762	1748507
Fatty Acid	Eicosadienoic acid (20:2)	3019646	2170967	2973664	1571384	1821420	1783456	3261100	5995832	11659270	21501402	3201022	11778914
Fatty Acid	Dihomo-linolenate (20:3n6)	4981649	5165394	4900756	3446450	4683447	3261884	3189852	6923204	11014112	19553901	3245276	11780730
Fatty Acid	Arachidonate (20:4)	58121678	43982532	48718048	48316426	53205502	32009486	38147965	62753460	92257667	111152466	54370862	80700296
Fatty Acid	Eicosapentaenoic acid(20:5 n3)	11973520	26753854	10155856	4796376	8131484	6229532	6261192	12161061	19073047	13185170	1984482	9702012
Fatty Acid	Erucic acid(22:1)	172	98	304	386	566	208	180	178	485	191	164	119
Fatty Acid	Docosatrienoic(22:3)	45080	36296	43537	20454	29008	29188	43886	94675	114482	277854	41866	136658
Fatty Acid	Adrenate (22:4n6)	1902598	1391900	2116610	1514268	2090412	1238188	2277608	5275424	8054188	15183650	3235374	8498164
Fatty Acid	Docosahexaenoic acid(22:6;4,7,10,13,16,19)	66156898	56097094	66258799	50869751	58129380	39113974	21154316	41022692	50499194	72729799	31576549	46959814
Lyso PC	1-Myristoylglycerophosphocholine(14:0)	1932	1246	1634	1860	1600	1356	804	766	1008	1746	1032	2589
Lyso PC	1-Eicosapentaenoyl-glycero-3-phosphocholine(20:5)	30	20	15	16	13	30	18	18	0	14	27	12
Lyso PC	1-Linoleoylglycerophosphocholine(18:2)	21	25	46	90	80	85	38	42	82	126	52	178
Lyso PE	Lyso PE(18:0)	740	661	1230	4834	4205	3608	5726	1758	2502	1884	1386	1790
Phosphatidyl glycerol	PG(18:1)	266	232	438	1166	1335	754	919	718	1804	580	486	362
Phosphatidyl inositol	PI(18:1)	2830	1386	2820	8966	14154	8986	2854	1630	3890	1584	1277	1024
Phosphatidylcholine	PC(C16/2:0)	3615	3326	4470	18588	22884	17724	41296	14602	18752	12746	8840	7612
Phosphatidylcholine	PC(28:0)	47	25	51	210	280	238	252	93	223	81	0	0
Phosphatidylcholine	PC(30:0)	801	577	927	3412	4869	3720	3423	1513	2088	1426	1138	457
Phosphatidylcholine	PC(32:0)	24856	16958	29430	104758	132069	108271	118172	47042	68522	47204	34578	11372

Phosphatidylcholine	PC(32:1)	16842	11178	11702	46602	94848	69292	80776	24679	59836	22888	13884	4762
Phosphatidylcholine	PC(34:1)	208902	126249	213588	593818	621625	627856	761173	357858	450346	329302	299903	132262
Phosphatidylcholine	PC(34:2)	354525	297611	397126	965286	961678	980074	1005049	496682	621910	428037	458603	259566
Phosphatidylcholine	PC(34:3)	11562	7231	9675	30078	40873	36990	69031	27756	61964	29984	19765	7774
Phosphatidylcholine	PC(36:0)	130	83	158	573	1134	712	1584	466	1628	508	220	81
Phosphatidylcholine	PC(36:1)	28167	9262	23532	101476	212464	136006	194575	59424	164810	74095	29498	9631
Phosphatidylcholine	PC(36:2)	262457	148372	260130	770127	843912	788334	729959	329820	485761	307252	218325	84928
Phosphatidylcholine	PC(36:3)	141148	55021	129720	417824	527998	371397	392554	152148	295652	140276	77117	35123
Phosphatidylcholine	PC(36:3)	2846	1249	2028	8139	13990	9960	17148	6148	16784	5760	3048	1087
Phosphatidylcholine	PC(36:4)	1971	0	0	6978	10186	5541	6598	2285	7458	2974	1540	6968
Phosphatidylcholine	PC(36:4)	198040	140270	214078	588840	602053	598181	878082	458462	496558	334232	332471	145775
Phosphatidylcholine	PC(36:5)	25409	27324	30427	60396	40707	57746	52594	32224	23144	26784	40114	27301
Phosphatidylcholine	PC(36:5)	6034	4570	8322	31274	56200	26780	19358	5706	21844	5662	2442	928
Phosphatidylcholine	PC(38:2)	2344	972	2220	9643	16746	7167	13372	4092	12583	3946	1738	470
Phosphatidylcholine	PC(38:3)	8020	4825	6678	34264	69688	39620	95190	31101	62200	21095	8362	2661
Phosphatidylcholine	PC(38:4)	1564	456	884	10848	18814	9805	39028	11100	28275	11230	2933	1008
Phosphatidylcholine	PC(38:4)	3073	2275	3191	8144	6702	7556	28022	4714	4958	3638	3807	1864
Phosphatidylcholine	PC(38:5)	41981	22227	38530	158128	209600	143496	329194	126090	213479	119068	68136	26477
Phosphatidylcholine	PE(38:6)	33	0	43	160	275	132	326	172	509	65	55	0
Phosphatidylcholine	PC(38:6)	8670	5782	12456	54585	90795	43054	121548	37190	94678	27382	10096	3586
Phosphatidylcholine	PC(38:6)	223772	169716	248915	630882	642995	640672	832124	415448	486908	316886	301748	110544
Phosphatidylcholine	PC(40:4)	348	166	461	2147	3926	2235	7522	2076	7234	2574	1020	346
Phosphatidylcholine	PC(40:4)	88	76	118	340	541	349	753	342	732	278	119	35
Phosphatidylcholine	PC(40:5)	499	0	0	1896	2636	2088	18264	6211	14374	8676	3406	1118
Phosphatidylcholine	PC(40:5)	3187	1730	2991	12502	17650	11970	13867	4962	9864	5619	3358	1148
Phosphatidylcholine	PC(40:6)	88522	43661	82069	288044	403735	339203	475701	195226	298960	152543	94008	23526
Phosphatidylcholine	PC(40:7)	510	266	580	2866	3857	2149	3280	1025	2232	752	386	156
Phosphatidylcholine	PC(40:7)	23968	13209	21766	97068	124364	80452	175538	65750	125051	51970	29294	8819
Phosphatidylcholine	PC(40:8)	152	156	190	556	494	598	2396	1130	1170	1056	961	470
Phosphatidylcholine	PC(48:2)	215	87	108	446	556	340	236	178	552	206	151	36
Phosphatidylethanolamine	PE(32:2)	100	44	80	216	349	258	198	93	193	60	0	0
Phosphatidylethanolamine	PE(34:0)	66	48	82	253	433	358	632	220	360	184	98	39

Phosphatidylethanolamine	PE(34:1)	1842	1200	2050	5438	8312	7346	11132	3769	7468	4584	2776	1302
Phosphatidylethanolamine	PE(34:2)	39152	24798	37610	109444	135792	121158	112396	46512	86358	45011	30068	11660
Phosphatidylethanolamine	PE(34:3)	1953	1040	1320	4893	8100	5598	3571	1323	3790	1010	685	294
Phosphatidylethanolamine	PE(36:0)	23	25	32	84	112	98	160	64	109	63	37	0
Phosphatidylethanolamine	PE(36:2)	56192	23894	53884	150851	199440	170926	124713	48221	101672	57208	30375	11976
Phosphatidylethanolamine	PE(36:4)	26947	17681	28386	88770	87624	83190	120692	58992	75366	45811	33530	13628
Phosphatidylethanolamine	PE(38:4)	71452	50396	85214	209650	215552	223285	315043	153649	188026	124172	110822	46548
Phosphatidylethanolamine	PE(40:6)	60346	33750	65899	200679	250679	221775	293284	133692	206835	110539	62936	17758
Prostaglandins	Prostaglandin E2	12	24	21	0	0	31	46	26	31	24	42	16
Prostaglandins	Prostaglandin B2	32	29	20	24	14	52	64	36	42	16	32	12
Prostaglandins	Prostaglandin I2	768	826	742	923	784	905	1232	772	770	596	1094	814
Sphingolipids	SM(d18:1/16:0)	9722	7816	11182	49386	63532	46894	71764	30386	37528	25808	21778	10103
Steroids	Cholesterol sulfate	334	294	338	710	829	670	1215	790	1138	650	570	423
Steroids	Estriol	28	28	38	34	46	25	44	16	28	28	60	26
Sterol metabolism	CE(16:0)	26315	8523	30334	59835	75320	57814	88830	61956	114111	52972	36998	5309
Sterol metabolism	CE(17:0)	212	67	242	578	518	509	710	492	1248	499	280	29
Sterol metabolism	CE(18:0)	518	149	754	1256	1764	1396	1988	1652	4159	1936	938	118
Sterol metabolism	11beta-Hydroxyandrost-4-ene-3,17-dione	35	22	36	36	32	48	20	0	0	15	0	7
Triacylglycerol	TG(58:10)	75664	32220	100321	254378	231422	177018	20752	25662	46097	23979	14692	0
Triacylglycerol	TG(46:3)	606	318	274	940	1250	1083	482	382	1086	339	261	522
Triacylglycerol	TG(52:6)	72874	62981	32419	114798	173869	157206	116704	116048	220346	61954	56878	6781
Triacylglycerol	TG(44:1)	5634	3204	2282	7366	11840	13302	4494	3483	10558	5049	3515	652
Triacylglycerol	TG(58:10)	5408	2770	7128	18674	21636	16092	13042	15266	23259	10203	7730	1042
Triacylglycerol	TG(54:7)	64409	93090	53232	172709	170216	185384	132066	118124	131180	46286	45936	3652
Triacylglycerol	TG(48:3)	72594	47815	51172	138194	155229	160802	68848	48287	93914	65550	57440	7370
Triacylglycerol	TG(50:4)	133952	113224	105246	269564	286759	294383	191812	134952	208193	110748	120730	24246
Triacylglycerol	TG(52:5)	168127	149068	135960	338250	348200	333892	252470	192236	276490	131766	148670	40470
Triacylglycerol	TG(52:6)	2728	2660	3036	10070	10214	11864	6544	5498	6490	4917	4583	998
Triacylglycerol	TG(54:6)	273980	163360	238566	550214	571132	511906	254632	191137	320700	210230	206336	53204
Triacylglycerol	TG(58:9)	50566	22172	70683	171034	143202	119397	29697	29048	36778	30018	22792	5186
Triacylglycerol	TG(56:8)	74946	78904	89774	259388	231374	228100	138292	125032	121924	71543	72471	17082
Triacylglycerol	TG(51:4)	31441	18177	20190	59105	67141	49071	20634	16203	27766	13075	10668	2182

Triacylglycerol	TG(56:7)	118737	76100	104582	289831	296080	249059	162309	134336	192534	116096	101835	21043
Triacylglycerol	TG(54:6)	87140	90488	77246	219179	226891	224478	303346	236530	260352	126156	134866	26192
Triacylglycerol	TG(58:8)	24890	10124	27363	75188	71167	49568	12960	14752	20042	13656	10952	1764
Triacylglycerol	TG(52:5)	1704	1944	1647	5531	5078	5922	3102	2525	0	1530	1206	0
Triacylglycerol	TG(56:8)	497	216	890	1204	1071	914	374	369	467	188	144	42
Triacylglycerol	TG(58:9)	370	82	630	698	626	454	0	0	0	0	0	0
Triacylglycerol	TG(48:2)	59118	52084	51333	135314	139852	162770	88352	54823	67084	57752	63041	16484
Triacylglycerol	TG(50:3)	153530	163603	128066	341024	355584	386891	340390	205660	246292	148862	197683	72528
Triacylglycerol	TG(54:5)	271940	148994	265258	574870	557192	517550	311375	214278	316290	229458	243588	103914
Triacylglycerol	TG(58:8)	21150	9135	30136	74939	67758	56721	16458	14969	18124	16318	11940	3937
Triacylglycerol	TG(56:7)	39457	36410	43702	130456	125419	120136	91645	77787	82054	54648	57790	18507
Triacylglycerol	TG(52:4)	364148	365880	310602	810125	793981	829310	952768	625064	765335	421620	546814	223813
Triacylglycerol	TG(46:1)	74	66	62	191	152	288	136	0	126	98	144	120
Triacylglycerol	TG(51:3)	63076	41128	38965	118682	132688	109168	71468	56086	75900	37118	36280	6860
Triacylglycerol	TG(58:7)	19916	7518	19461	56835	67169	38792	18720	21296	30672	20898	15212	2694
Triacylglycerol	TG(56:6)	81118	55868	69106	214984	234887	187720	205682	183810	235516	135090	120382	23196
Triacylglycerol	TG(54:5)	58072	46872	44532	145525	165596	152044	217258	168380	194188	105266	110126	21853
Triacylglycerol	TG(52:4)	7142	6198	5816	21423	23073	25266	15806	11372	12855	10160	9355	1940
Triacylglycerol	TG(50:2)	147324	148364	123232	343890	367928	421794	298365	179625	207079	136125	173546	72026
Triacylglycerol	TG(48:1)	22342	21541	20122	58925	60728	78340	34972	19394	19924	22072	25551	8822
Triacylglycerol	TG(52:3)	359540	346013	315865	826612	802370	836046	928726	602754	744418	415305	534220	268435
Triacylglycerol	TG(54:4)	260117	131148	243290	539590	524804	488054	314136	223017	339227	236856	239571	109473
Triacylglycerol	TG(56:5)	48240	18068	50096	116336	130815	82876	69388	79306	113507	53232	31505	8056
Triacylglycerol	TG(56:6)	4751	3399	5064	17692	22408	23667	6276	6116	11624	9262	7140	1272
Triacylglycerol	TG(58:7)	8563	4218	10282	30770	34208	25214	6682	7468	12883	7550	5620	1400
Triacylglycerol	TG(58:6)	10563	4046	10034	30694	39993	23092	14694	19351	34720	16260	10768	1790
Triacylglycerol	TG(46:0)	26	73	62	116	100	110	106	40	0	40	72	100
Triacylglycerol	TG(53:3)	83848	32126	51031	153320	187575	125940	78270	74706	144397	59876	46066	7382
Triacylglycerol	TG(54:4)	10120	6088	7805	32630	40476	31214	26271	29936	28877	16177	10138	1450
Triacylglycerol	TG(56:5)	22896	16099	18398	71768	85534	58430	66331	72488	100902	47665	47392	4856
Triacylglycerol	TG(52:2)	255338	246714	219498	591782	599934	639286	769744	518920	640470	352098	436038	202153
Triacylglycerol	TG(48:0)	2095	1826	2258	7324	7630	10792	2517	1298	1158	1712	1810	1054

Triacylglycerol	TG(50:1)	68280	57312	62670	173712	185434	218432	98046	57767	73247	59892	66986	32554
Triacylglycerol	TG(54:3)	214810	107915	189758	435424	443644	395965	273222	198686	317300	202868	202063	85416
Triacylglycerol	TG(56:4)	48796	15978	56176	108912	118584	66966	25116	27246	47129	22346	18321	5080
Triacylglycerol	TG(51:1)	15163	4122	7298	26832	48532	31766	13038	14730	40076	14476	8042	948
Triacylglycerol	TG(53:2)	41754	10718	21815	71136	115201	64391	41710	50050	132385	37278	22413	3302
Triacylglycerol	TG(58:5)	1064	534	1000	3506	5545	2410	1276	1808	6066	1443	858	141
Triacylglycerol	TG(56:4)	1112	610	1132	3880	6560	4388	2336	3150	11616	3970	1785	275
Triacylglycerol	TG(52:1)	48162	27098	36788	107770	139660	153117	101154	88696	127328	84496	71110	13180
Triacylglycerol	TG(56:3)	61755	15692	54207	106572	141492	73949	33606	36820	76026	28680	21026	3791
Triacylglycerol	TG(54:2)	112683	47708	87781	213176	274305	213807	165876	158363	245068	120647	97836	19380
Triacylglycerol	TG(50:0)	2287	1530	2260	8025	10061	13172	5058	4334	5738	5274	3617	801
Triacylglycerol	TG(58:4)	6467	1488	7637	13460	17982	7956	1876	2422	4873	1940	1557	0
Triacylglycerol	TG(53:1)	3825	782	2436	7015	13396	7361	2397	2481	9532	3138	1559	257
Triacylglycerol	TG(51:0)	302	114	272	854	1328	975	336	314	792	391	252	66
Triacylglycerol	TG(56:2)	12488	2666	9823	16955	37538	16928	9182	14380	39291	8430	4564	694
Triacylglycerol	TG(58:3)	6555	1154	6000	9993	20990	8200	1878	2774	10686	2359	1313	221
Triacylglycerol	TG(54:1)	6985	2116	5474	11027	25374	15822	13282	20190	42699	11676	5816	818

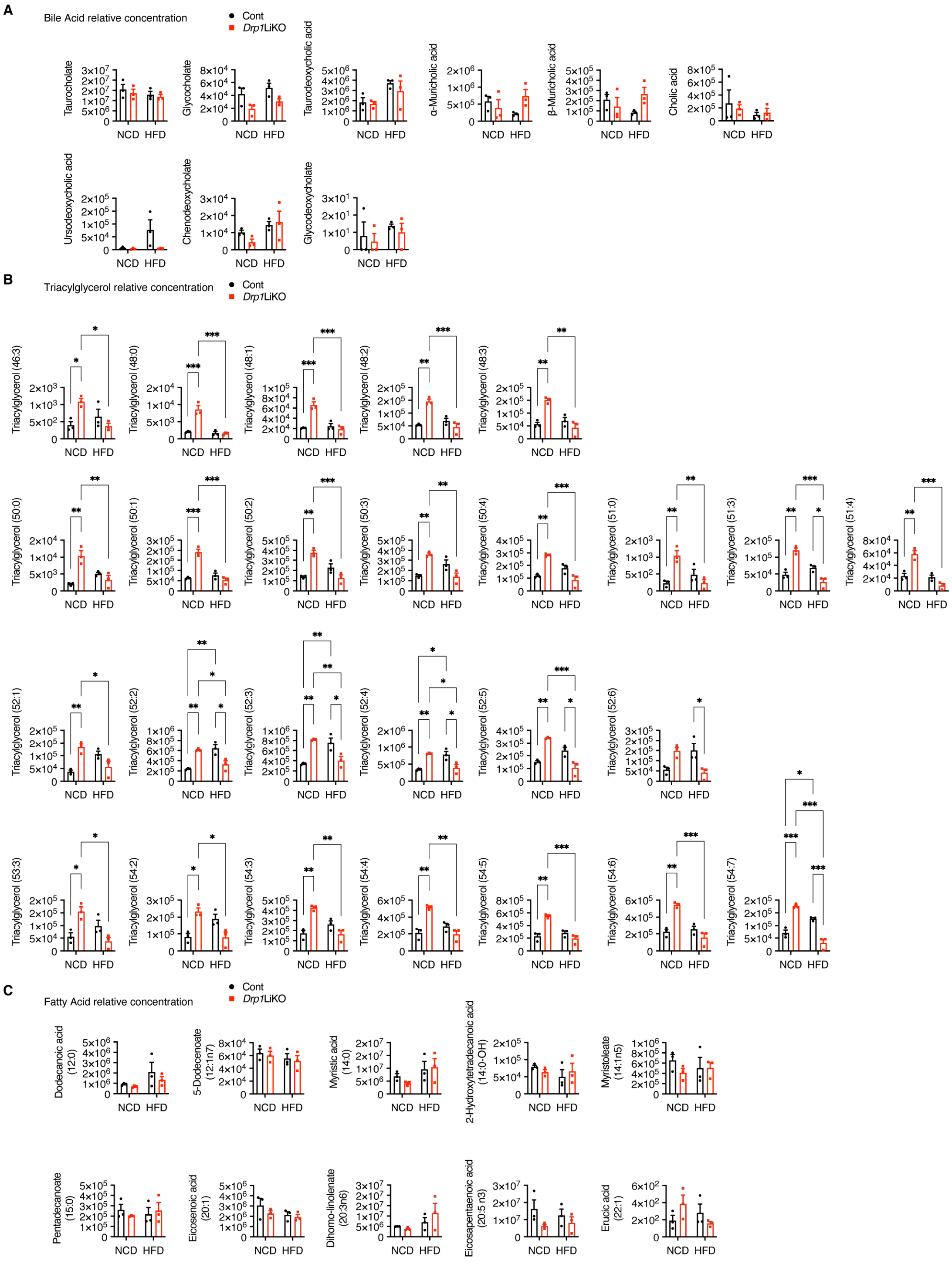

Supplementary Figure 1

Supplementary Figure S1. Identified fatty acid metabolic pathways in HFD-*Drp1*LiKO mice. Relative concentrations of bile acid (**A**), triacylglycerol (**B**), and free fatty acid (**C**) in the livers of control and *Drp1*LiKO mice. The quantities of triacylglycerol and free fatty acids are categorized by total carbon

atoms and the number of double bonds. Values expressed as mean ± SEM. $n = 3$. $^{*}p < 0.05$, $^{**}p < 0.01$, $^{***}p < 0.001$. Determined by two-way ANOVA with Tukey's multiple comparisons test.

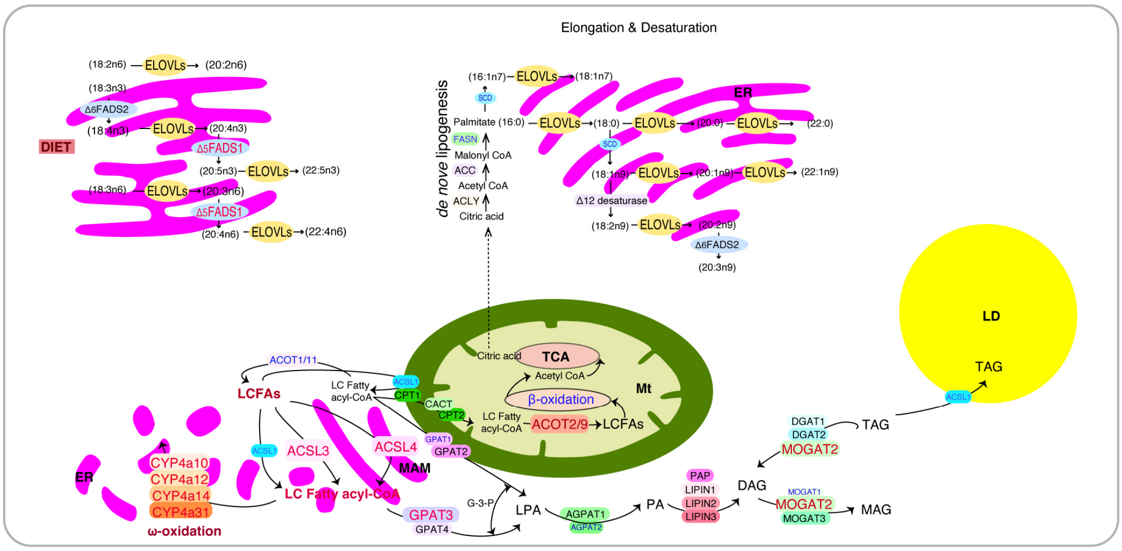

Supplementary Figure 2

Supplementary Figure S2. Signaling pathway of differentially expressed lipid metabolism-associated genes in *Drp1*LiKO mice. Several genes related to fatty acid biosynthesis and mitochondrial β-oxidation demonstrated reduced expression in HFD-*Drp1*LiKO mice. Genes encoding cytochrome P450, family 4, subfamily a (*Cyp4a*), which are vital enzymes involved in microsomal ω-oxidation, exhibited elevated expression levels. Genes encoding ELOVL fatty acid elongase (*Elovl*), and fatty acid desaturase (*Fads*), which are involved in fatty acid transport, elongation, and desaturation, exhibited increased expression levels. The gene encoding stearoyl-coenzyme A desaturase (*Scd*), which regulates the desaturation of saturated fatty acids (SFAs) to monounsaturated fatty acids (MUFAs), exhibited decreased expression levels. Long-chain fatty acyl-CoA synthetases (Acsl) exhibiting different regulation patterns depending on their location. The expression levels of *Acsl1* decreased, whereas those of *Acsl3* and *Acsl4* increased. Fatty acid acyl-CoA thioesterases (Acots), which hydrolyze long-chain fatty acyl-CoA into FFAs, also exhibited different regulation patterns. Acot2 and Acot9 localize to the mitochondrial

matrix, whereas Acot1 and Acot11 localize to the cytosol. The expression levels of *Acot1* and *Acot11* decreased, whereas those of *Acot2* and *Acot9* increased.

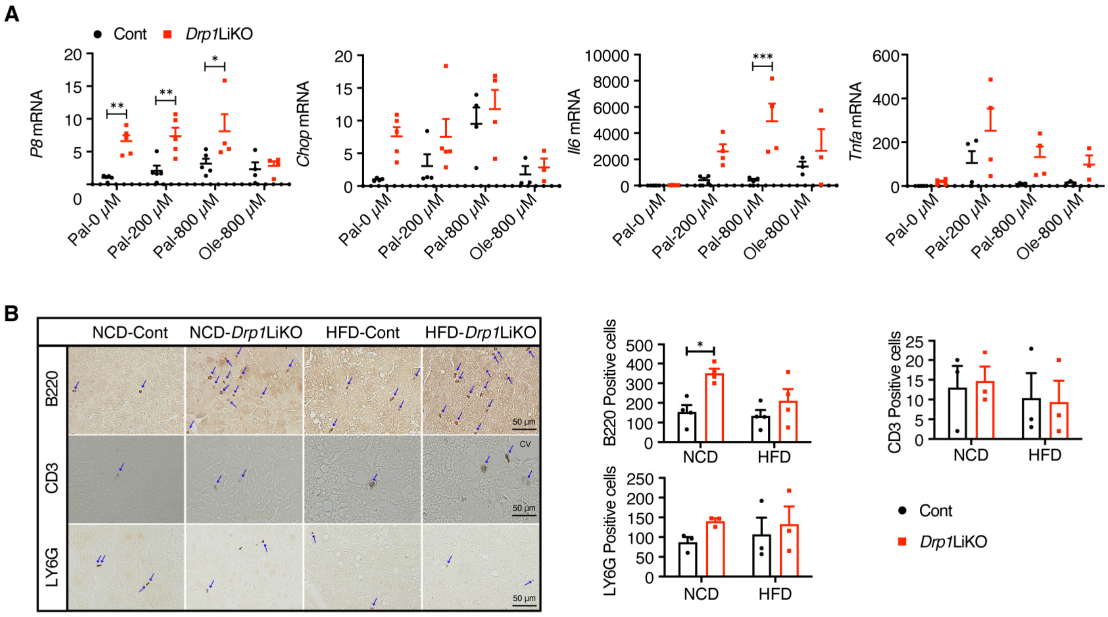

Supplementary Figure 3

Supplementary Figure S3. Inflammatory response in control and *Drp1*LiKO primary hepatocytes and mouse livers.

(**A**) Primary hepatocytes isolated from control and *Drp1*LiKO mice were treated with PBS (referred to as palmitate-0 μM), palmitate (200 μM or 800 μM), or oleate (800 μM) for 24 h. Expression of ER stress marker genes (*P8* and *Chop*) and inflammatory cytokines (*Il6* and *Tnfa*) was determined by quantitative real-time PCR. Results are normalized to *Gapdh* expression and shown as fold-changes relative to gene expression in PBS-treated control cells. Values are expressed as mean ± SEM (n = 3-5). *$p < 0.05$, **$p < 0.01$, ***$p < 0.001$ determined by two-way ANOVA with Sidak's post hoc test. (**B**) Representative images of CD3, Ly6G, and B220 staining in liver sections from either NCD or HFD fed control and *Drp1*LiKO mice. Scale bar = 50 μm. Quantitative analysis of the number of CD3-, Ly6G-, and B220-positive cells was performed by counting cells in 15 high-power fields (20×) per slide from 3-4 mice per group. Values are expressed as mean ± SEM (n = 3-4). NS, no significant difference; *$p < 0.05$ determined by two-way ANOVA with Tukey's post hoc test.

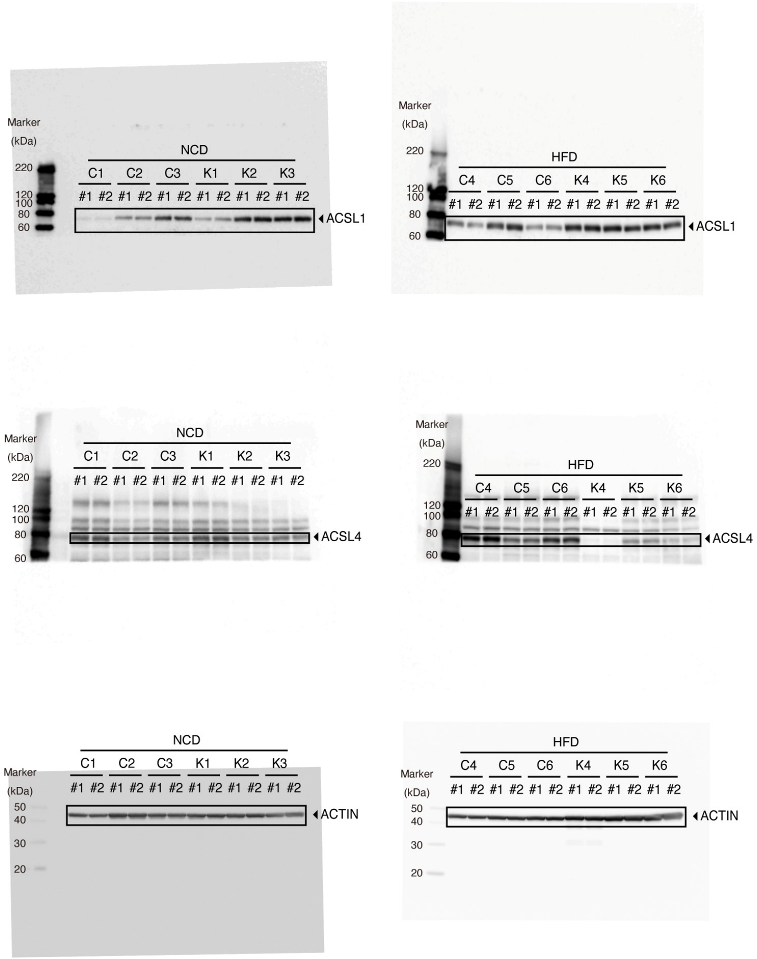

Supplementary Figure 4

Supplementary Figure S4. Uncropped western blot images for Figure 1E.

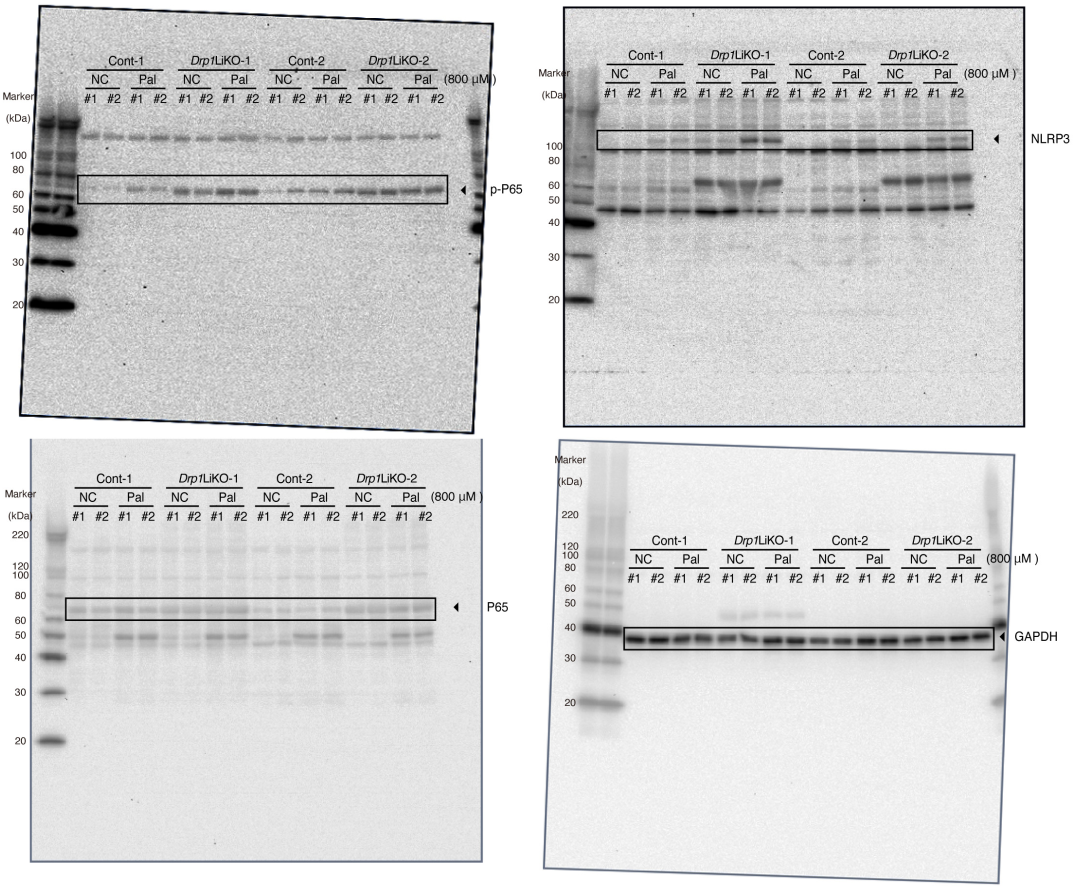

Supplementary Figure 5

Supplementary Figure S5. Uncropped western blot images for Figure 5B.
